# Modulation of *in vitro* Brain Endothelium by Mechanical Trauma: Structural and Functional Restoration by Poloxamer 188

**DOI:** 10.1038/s41598-020-59888-2

**Published:** 2020-02-20

**Authors:** Edidiong Inyang, Vinay Abhyankar, Bo Chen, Michael Cho

**Affiliations:** 1 Department of Bioengineering, University of Texas at Arlington, Arlington, TX United States; 2 Department of Biomedical Engineering, Rochester Institute of Technology, Rochester, NY United States

**Keywords:** Blood-brain barrier, Biomedical engineering

## Abstract

Brain injuries caused by an explosive blast or blunt force is typically presumed to associate with mechanical trauma to the brain tissue. Recent findings from our laboratory suggest that shockwaves produced by a blast can generate micron-sized bubbles in the tissue. The collapse of microbubbles (i.e., microcavitation) may induce a mechanical trauma and compromise the integrity of the blood-brain endothelium (BBE). To test our hypothesis, we engineered a BBE model to determine the effect of microbubbles on the structural and functional changes in the BBE. Using monolayers of mouse primary brain microvascular endothelial cells, the permeability coefficient was measured following simulated blast-induced microcavitation. This event down-regulated the expression of tight junction markers, disorganized the cell-cell junction, and increased permeability. Since poloxamers have been shown to rescue damaged cells, the cells were treated with the FDA-approved poloxamer 188 (P188). The results indicate P188 recovered the permeability, restored the tight junctions, and suppressed the expressions of matrix metalloproteinases. The biomimetic interface we developed appears to provide a systematic approach to replicate the structure and function of BBE, determine its alteration in response to traumatic brain injury, and test potential therapeutic treatments to repair the damaged brain endothelium.

## Introduction

Traumatic brain injury (TBI) is one of the major causes of emergency visits and hospitalization. In 2010, the Centers for Disease Control reported about 2.5 million emergency department visits, hospitalizations, and deaths here in the United States alone^1^. TBIs are also caused by an explosive blast or blunt force to the head especially among those who serve in the U.S. military^2–4^. The trauma can lead to endothelial cell detachment, tight junction disruption, and altered blood-brain barrier (BBB) permeability^5,6^. One of the unique features of BBB is the regulation of biotransport through a monolayer of brain endothelial cells (BECs). BECs are highly regulated in their structure and function by the tight junction complex that is composed of, among many molecules, zonula occludens (ZO-1) and the occludins family^7,8^. Only particles with a molecular mass of less than 500 Daltons can cross the BBB efficiently^9^. However, the structural integrity of the BBB can be mechanically and biochemically compromised^10–13^, allowing harmful substances to extravasate into the brain. The leaky brain endothelium, in turn, may lead to secondary brain injury^14–16^.

Several sophisticated TBI models of explosive blast or blunt force have been studied. However, the potential mechanisms connecting shock wave exposure to the head and to TBI are still not well understood^17^. One of the mechanical traumas that have been investigated in our laboratory is the formation of micron-size bubbles in response to shock wave and subsequent collapse of such microbubbles, referred to as microcavitation^18^. The collapse of highly pressurized microbubbles is thought to produce shear stress that can disrupt the brain endothelium^19,20^. Indeed our previously published work demonstrated that microcavitation detaches cultured cells from the substrate and creates an area that is devoid of cells. We coined the term “2D crater” to describe this microcavitation-induced damaged area. Although we initially documented the effect of microcavitation using astrocytes^18,21^, it is indeed interesting to investigate whether the microcavitation mechanism is responsible for disruption of the brain endothelium. Since a monolayer of mouse brain endothelial cells has been demonstrated to resemble the *in vivo* BBB phenotype, express excellent characteristics of the BBB, and form the functional barriers^22^, it offers a model system to elucidate the potential damage mechanisms that are associated with microcavitation.

Although brain trauma is increasingly better understood, it nonetheless remains elusive whether reparative treatments are plausible. This is rather important because a recent study suggests that approximately 320,000 soldiers may have experienced mild TBI during the Iraq and Afghanistan wars and that such injuries most often lead to cognitive degeneration and post-traumatic stress disorder^23^. However, there are only a limited number of therapeutic treatments currently available, and in most cases, they are confined to identification and treatment of only the symptoms. Pharmacological selective serotonin reuptake inhibitors, for example, have been approved by FDA, and some non-pharmacological treatments such as cognitive behavioral therapy may also be effective^24^. In addition, the use of a family of copolymers referred to as poloxamers offer an intriguing potential to mitigate the blast-induced cell damage^25–29^. Many studies have shown that poloxamers are capable of sealing the compromised cell membrane. For example, the FDA-approved poloxamers P188 was demonstrated to reconstitute the membrane in BBB^30,31^ and down-regulated the secretion of matrix metalloproteinases (MMP)^32,33^ by likely modulating the TNF-α pathway^34^. In this study, we cultured a monolayer of brain endothelial cells on a well-characterized synthetic membrane and quantitatively determined changes in the permeability and disorganized tight junctions in response to the blast-induced microcavitation. Our results show that microcavitation functionally and mechanically disrupts the BECs, and that treatment of brain endothelial cells with P188 mitigates the BBE disruption by alleviating the loss of tight junctions.

## Results

**Figure 1:**
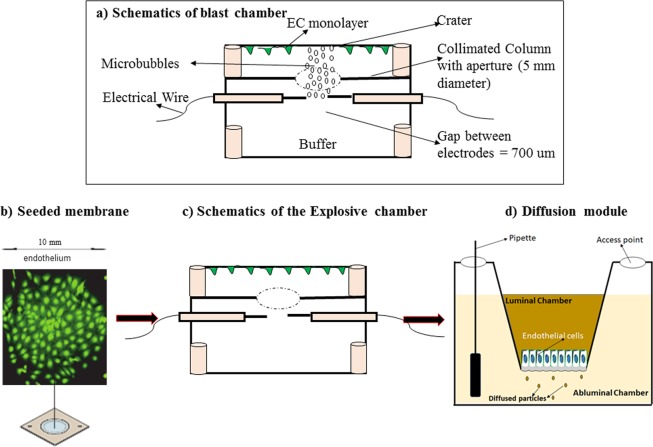
Schematics of the custom-designed blast chamber and a brief flow of experimental protocol from culture insert, proof of cell adhesion to PETE membrane to the blast chamber and finally the diffusion chamber. (**a**) The blast chamber was engineered to generate shockwave-induced microbubbles. They can only rise to the top of the chamber and collapse onto the seeded BECs, detaching cells from a controlled area referred to as a “crater”. (**b**) Cell culture insert. Green FITC cell tracker was used to demonstrate that the PETE membrane coated with fibronectin supports endothelial cell cultures. (**c**) Diagram representation of the blast chamber that highlights an aperture to control the formation of a single crater that can be tracked and monitored. (**d**) Schematic description of the diffusion chamber with a monolayer of cells on the luminal side of the membrane. Permeability was measured by introducing FITC dextran dye of different molecular weights into the luminal chamber and measuring the time-dependent concentration in the abluminal chamber.

A schematic drawing of the microcavitation/diffusion chamber is shown in Fig. 1. We have used the chamber to study the effects of microcavitation and have reported the results in detail elsewhere. Prior to cell culture, a synthetic polyethylene terephthalate (PETE) membrane (1 um diameter pores) was coated with fibronectin (1 ug/ml). The insert that contains a monolayer of endothelial cells allowed easy handling between the two chambers to expose the cells to microbubbles first (Fig. 1a) and then perform the permeability measurements. To establish the PETE membrane supports cell culture, BECs were pre-incubated with a cell tracker (green; Fig. 1b) for 30 minutes before seeding on the membrane and shown to reach confluence at day 4. The insert was placed in the microcavitation chamber (Fig. 1c) and then moved to measure the permeability coefficient (Fig. 1d).

**Figure 2:**
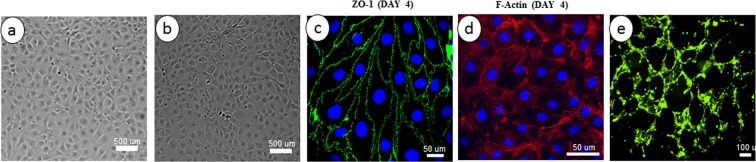
Characterization of mouse primary brain microvascular endothelial cells (MPBMECs). (**a**) Phase contrast microscopic view of the MPBMECs showing a tightly packed cell organization. (**b**) Human umbilical vascular endothelial cells (HUVECs) demonstrate a similar morphology. (**c**) Immunofluorescence staining demonstrating the expression of endothelial cell tight junction-associated marker, ZO-1 (green) after 4 days of culture. (**d**) Immunofluorescence staining of mouse endothelial cell F-actin (red) and nuclei counterstained with DAPI. (**e**) Branched network of capillary-like cords formed by MPBMECs in Matrigel extracellular matrix after 6 hours in EGM-2 medium (X20). The scale bars are specified in each image panel.

The cells used in this study showed a morphology similar to that of primary cultures of brain endothelial cells and exhibited a monolayer of tightly packed elongated shape that demonstrated cell-cell contact at the confluence (Fig. 2a). At confluence, the cells also showed the spindle-shaped morphology that was previously documented in brain endothelial cells derived from human (Fig. 2b). The cells were also examined for the expression of tight junction protein ZO-1 (Fig. 2c) and F-actin stress fibers (Fig. 2d) at day 4 of culture. The BECs maintained a non-transformed phenotype over more than 6 passages without any sign of senescence. For example, when the cells from passage 5 or 6 were seeded on a reconstituted extracellular matrix (Matrigel), the cells rapidly formed a branched capillary-like cords network, which is characteristic of primary endothelial cells and suggests the normal capacity for angiogenesis (Fig. 2e).

**Figure 3:**
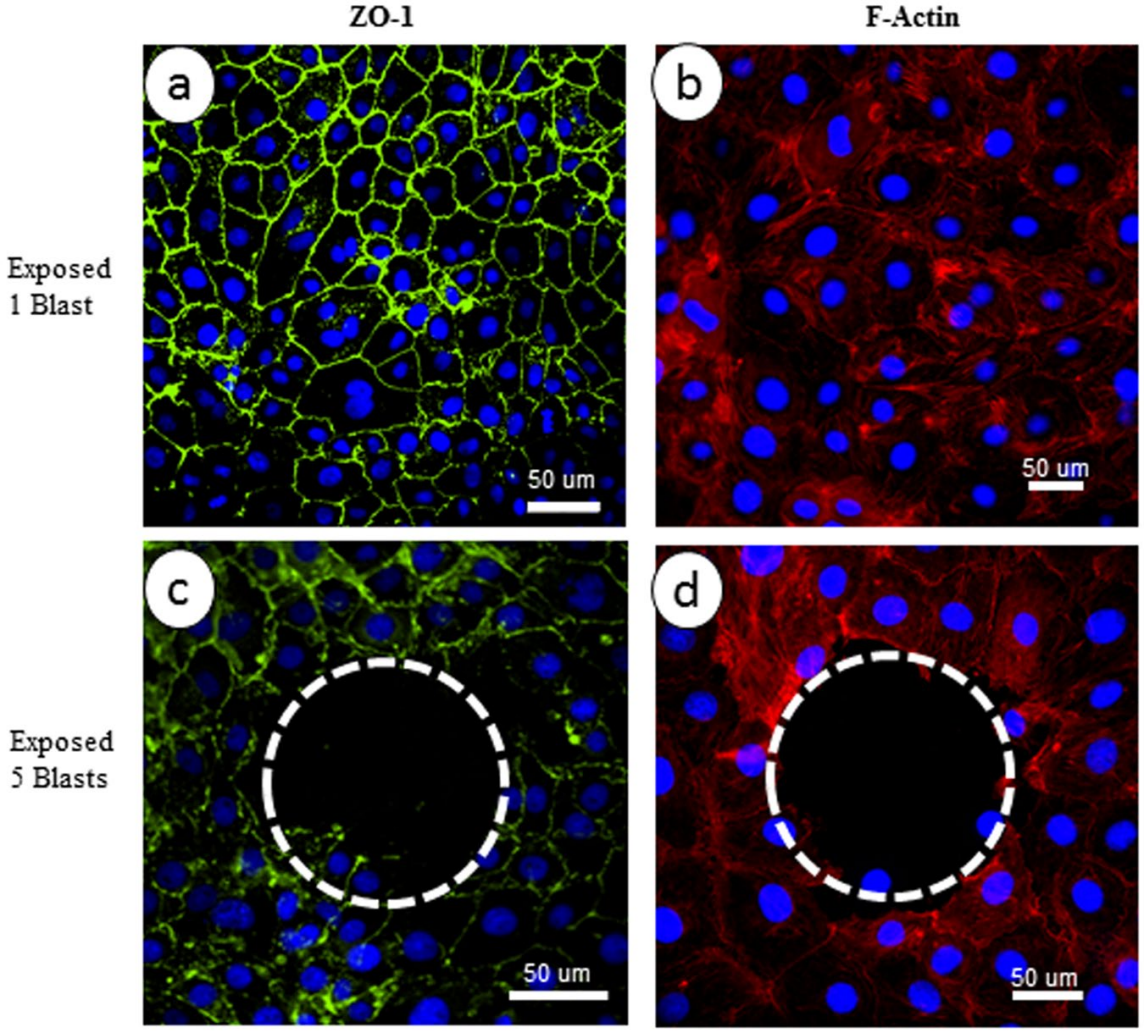
Disruption of blood brain endothelium. Immunofluorescence staining of endothelial cells for ZO-1 (green), F-actin (red) following microcavitation. (**a**,**b**) Exposure to one blast and (**c**,**d**) exposure to five repetitive blasts. There is no obvious disorganization of tight junctions in response to one blast exposure, whereas formation of the crater following five repetitive blasts is accentuated by the dotted circles. The nuclei (blue) were stained with DAPI. Bar = 50 μm.

One of the characteristics of physical phenomena we repeatedly observed and reported in detail is that the collapse of microbubbles creates a region of interest in which the cells are detached from the substrate. To further illustrate this phenomenon, the endothelial cells were exposed to microcavitation produced by one or five repetitive blasts. In response to a single blast, the cells remained intact, but the tight junctions and F-actins appeared disorganized and disrupted (Fig. 3a,b). In contrast, five repetitive blasts caused cell detachment and created a void as indicated by the dashed line circle (Fig. 3c,d). The region devoid of cells was referred to as a *2D crater*. We recently estimated that the collapse of microbubble can produce a pressure of ~55 kPa. These highly pressurized microbubbles can indeed produce a secondary shear stress (e.g., microjet) that is sufficient to detach cells and also impact the cells around the periphery of the crater.

**Figure 4:**
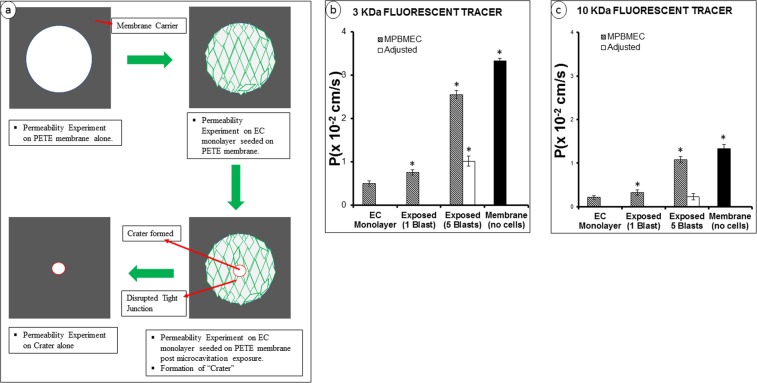
Schematics of measurement of the permeability coefficient (*P*). (**a**) Experimental protocols summarized for the determination of Dextran permeability across *in vitro* BBE model. **(b**,**c)** Permeability coefficients measured using 3 or 10 kDa Dextran molecules. The adjusted permeability coefficients were determined according to the procedures shown in (**a**). This was necessary to delineate the permeability through the crater and through the tight junctions. The adjusted permeability coefficients reflect the altered biotransport properties through tight junctions. Data represent mean ± SD from 6 independent experiments. *Indicates p < 0.05 when compared to the control (i.e., EC monolayer).

In order to appropriately assess the permeability, we carried out diffusion of two tracer molecules under various experimental conditions. Formation of a crater induced by microcavitation should significantly enhance the permeability and may overwhelm the subtle changes of diffusion through the disorganized tight junctions. Because the location and size of the crater are predictable, a simple microfabrication technique was applied to assess and delineate the diffusion through the 2D crater. The experimental procedures are schematically described in Fig. 4a. Using a PETE membrane, we blocked the 1 um PETER membrane pores except in an area of a 200 um diameter circle to mimic diffusion through the crater. Adjusted permeability coefficients (P_adj_) was then calculated by subtracting the permeability coefficient through the crater only without any seeded cells. This adjustment was not needed in response to a single blast (i.e., no crater formed) but was necessary to delineate diffusion through the disorganized tight junctions in response to five repetitive blasts. As expected, the largest permeability coefficient (3.33 × 10^−2^ cm/s) using 3 kDa Dextran was obtained using the PETE membrane alone without cell seeding (Fig. 4b). When a monolayer of endothelial cells was cultured on one side of the PETE membrane, the permeability of 3 kDa Dextran was measured to be more than 6-fold smaller (0.50 × 10^−2^ cm/s). In response to a single blast, the permeability was increased moderately but still statistically significant. However, when five blasts were applied, the unadjusted permeability of 3 kDa Dextran increased noticeably, but P_adj_ was determined to be 2-fold greater than the control value with seeded monolayer of endothelial cells. We performed similar diffusion experiments using 10 kDa Dextran tracers. The permeability coefficients measured were generally much smaller, as expected (Fig. 4c). The permeability of 10 kDa Dextran through the 1 um pores was about 3 times smaller than that using 3 kDa Dextran. When a monolayer of endothelial cells was cultured, the permeability coefficient of the larger Dextran again decreased from 0.50 × 10^−2^ cm/s (3 kDa) to 0.21 × 10^−2^ cm/s. In response to a single blast, the permeability coefficient remained unchanged (0.23 × 10^−2^ cm/s). Interestingly, a 5-blast exposure did not change the permeability of 10 kDa Dextran when adjusted for diffusion through the crater. The potential implication might be that even though the tight junctions are disrupted, the large 10 kDa molecules are still prevented from diffusing through the disorganized tight junctions.

**Figure 5:**
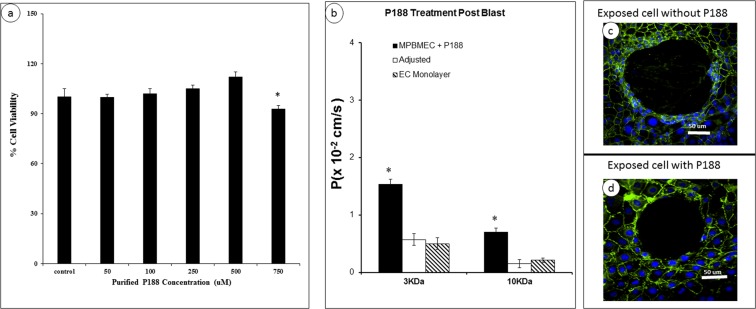
Dose dependent study of P188 on brain endothelial cell viability and modulated permeability coefficient. (**a)** Cell viability was measured after 24 h with MTT assay. Data represent mean ± SD from 6 independent experiments. *p < 0.05 when compared to the control (no P188 applied). **(b)** Changes in the permeability coefficients of the monolayer exposed to microcavitation and treated with P188 using 3 or 10 kDa Dextran molecules. *p < 0.05 compared to control (EC monolayer) from 6 independent experiments (n = 6). (**c**) Expansion of the crater if left untreated. Cells were exposed to microcavitation and incubated in serum-free media for 1 h and stained for ZO-1 (green) and nuclei (blue). (**d**) The crater expansion is prevented if the cells were treated with P188 (500 μm) for 6 hrs following microcavitation. Immunofluorescent images showed reconstitution of the tight junctions in response to P188.

Poloxamer P188 is a surfactant that was originally approved by the FDA as a blood-thinning substance. P188 has since been demonstrated to seal the membrane of damaged cells and repair the cellular function. In our previous studies, we have successfully applied P188 to reconstitute the viability and functionality of astrocytes by restoring the calcium dynamics and minimizing oxidative stress. Prior to applying P188 for potential restoration and repair of the damaged BECs, we first determined the dose dependence of P188 by varying its concentration from 0 to 750 uM and tested the cell viability using MTT assay (Fig. 5a). After 24 hours, P188 did not change the cell viability up to the 0.5 mM concentration. It should be also noted that P188 can form micelle at ~1 mM concentration. Based on these findings, we chose to apply P188 (0.5 mM) to the injured endothelial cells and determined potential beneficial effects by measuring the permeability coefficient and examining tight junctions (e.g., ZO-1 gene expression). The Dextran permeability was substantially decreased when the cells were exposed to a 5-blast microcavitation event and then treated with P188 for 6 hrs (Fig. 5b). When adjusted for diffusion through the 2D crater, the reduced permeability was proven statistically significant. The P188 reduced the permeability coefficient of 3 kDa Dextran to 0.57 × 10^−2^ cm/s. This value is essentially indistinguishable from the experimental condition of using a monolayer but without exposure to microbubbles (0.50 × 10^−2^ cm/s). It is interesting to observe that, without treatment of P188, the 2D crater that was created by microcavitation seemed to expand (Fig, 5c) in comparison to when the sample was treated with P188 (0.5 mM) for 6 hrs (Fig. 5d). Taken collectively, the P188 treatment appears to restore the biotransport properties and stablize the damaged area (i.e, crater).

**Figure 6:**
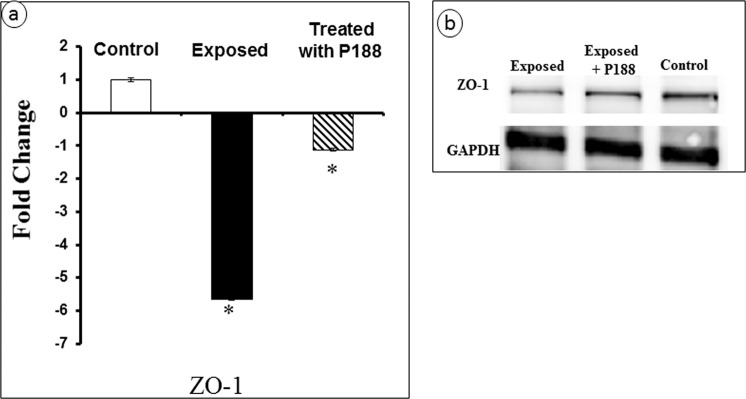
Gene expression and Western blot. **(a)** Change in the ZO-1 gene expression determined from 3 independent experiments (n = 3). *p < 0.05 when compared to the control. **(b)** Western blot showing recovery of the ZO-1 protein level in response to P188 treatment is consistent with the gene expression measurements.

The effect of microcavitation on tight junction was further probed by determining the ZO-1 gene expression. A 6-fold decrease was measured. In contrast, when P188 was applied for 6 hours following a microcavitation event, the ZO-1 expression was significantly restored (Fig. 6a). In addition to PCR experiments, we performed Western blot experiments for ZO-1 using BECs. The ZO-1 protein level was significantly down-regulated in response to microcavitation (Fig. 6b; for full Western blot see Supplementary Fig. S1). Consistent with the gene expression results, endothelial cells treated with P188 showed at least a partial restoration at the protein level as well.

**Figure 7:**
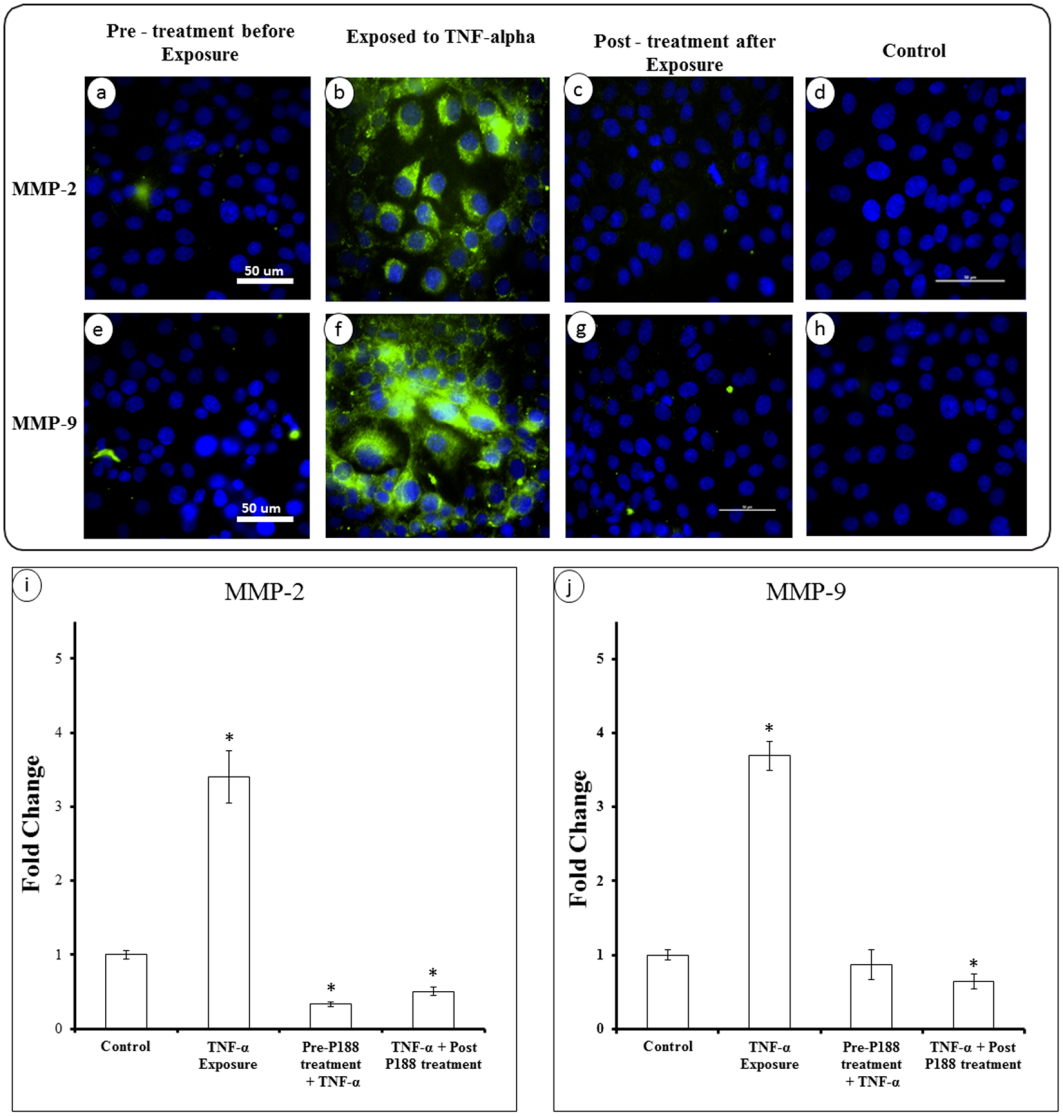
Inflammatory activation of MMPs and effect of P188. Immunofluorescent staining of MMP-2 & 9 in TNF-α activated endothelial cells. **(a**,**e)** BECs were pre-treated with P188 for 3 hrs prior to incubating with TNF-α for 1 hr and then stained for MMP-2 & 9, respectively. **(b**,**f)** BECs incubated with TNF-α for 1 hr no treatment with P188. **(c**,**g)** BECs incubated with TNF-α for 1 hr, post-treated with P188 for 3 hrs. **(d**,**h)** Images of MMP-2 & 9 in control cells with no treatment. The efficacy of P188 in suppressing the gene expression of MMP-2 & 9 in both pre-treated and post-treated groups. Fold change were calculated from triplicate experiments (n = 3). Data represent mean ± SD. *Indicates p < 0.05 when compared to the control (i.e., no P188 treated cells).

There are several studies in the literature that suggest a correlation between ZO-1 and matrix metalloproteinases (MMPs), specifically MMP-2 and MMP-9. We first introduced a chemical trauma (TNF-α) to endothelial cells. As shown in (Fig. 7d,h), there was essentially no expression of MMP-2 or MMP-9 in control cells. Treatment of cells with TNF-α induced measurable changes and upregulated both MMP-2 and MMP-9 (Fig. 7b,f). More interestingly, cells were found to down-regulate the MMPs if they were treated with P188 either before (Fig. 7a,e) or after (Fig. 7c,g) exposure to TNF-α. Fluorescent images indicate P188 indeed appears to effectively regulate the MMP expressions. To quantify the effect of P188 on the MMP expressions, we performed PCR experiments to determine the gene expression of MMP-2 and MMP-9 (Fig. 7i,j). Approximately a 4-fold increase in the MMP-2 and MMP-9 gene expressions were induced in response to TNF-α. Either pre- or post-treatment of cells with P188 mitigated the effect of inflammatory trauma and down-regulated the MMP expressions.

**Figure 8:**
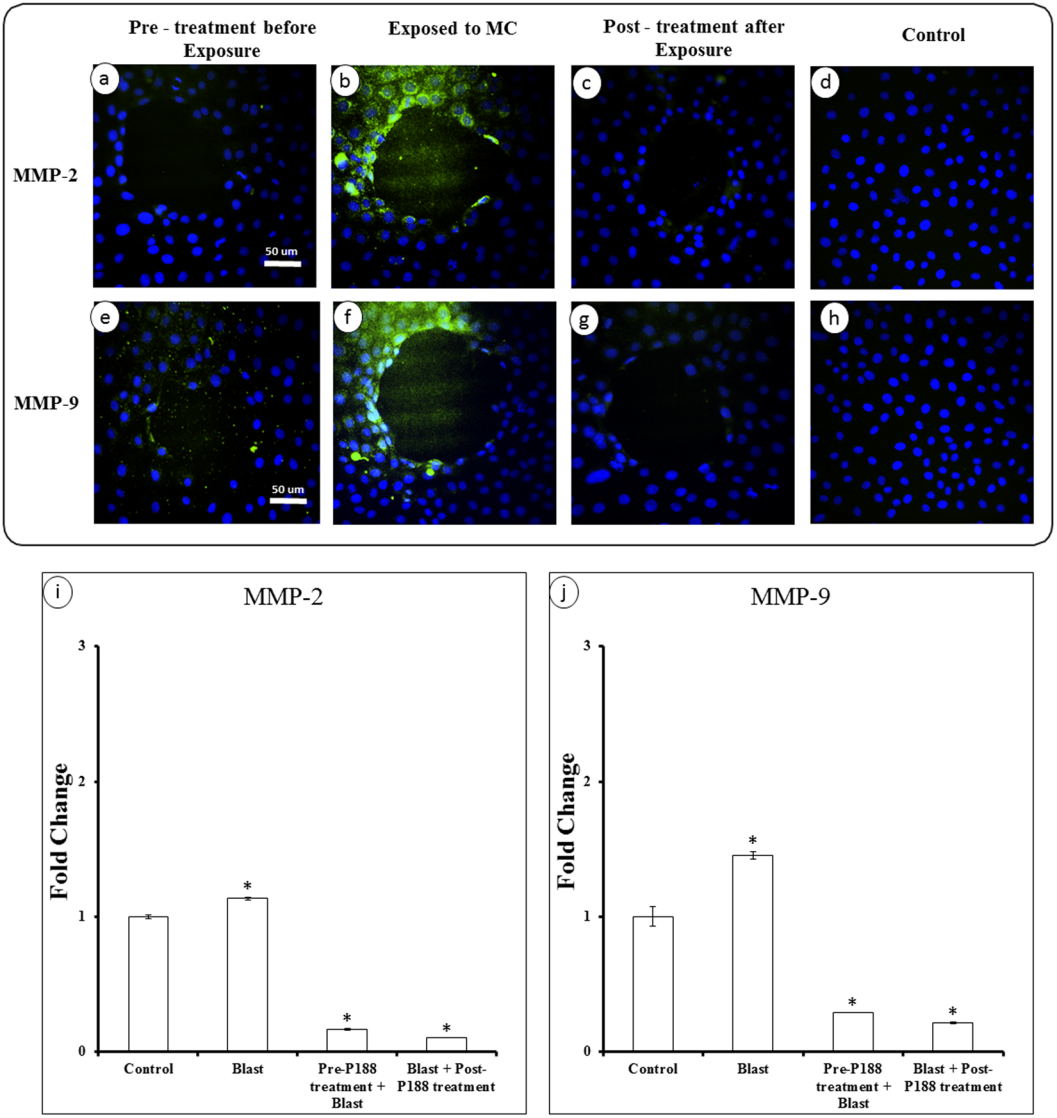
Mechanical activation of MMPs and effect of P188. **(a**,**e)** BECs were pre-treated with P188 for 3 hrs prior to exposure to microcavitation and then stained for MMP-2 & 9, respectively. **(b**,**f)** BECs were first exposed to microcavitation and then stained for MMP-2 & 9 without P188 treatment. **(c**,**g)** BECs exposed to microcavitation and post-treated with P188 for 3 hrs, and then stained for MMP-2 & 9. Results from all groups were compared to control **(d**,**h)**. **(i**,**j)** The efficacy of P188 in suppressing the gene expression of MMP-2 & 9 in both pre-treated and post-treated groups. Fold change were calculated from triplicate experiments (n = 3). Data represent mean ± SD. *Indicates p < 0.05 when compared to the control (i.e., no microcavitation and no P188 treated).

We next tested whether P188 can also minimize the MMP expressions in response to a mechanical trauma such as microcavitation. As illustrated in Fig. 8, we observed the similar results that the MMP expressions were up-regulated by microcavitation. However, the fluorescent image intensities would suggest the extent of the MMP upregulation was not as extensive as that induced by TNF-α. Nonetheless, treating the cells with P188 either prior to or following a microcavitation event was effective in significantly down-regulating the MMP expressions (Fig. 8a,h). Quantitative measurements of the MMP expressions are also consistent with the fluorescent images. However, unlike the 4-fold increase in the MMP gene expressions induced by TNF-α, the microcavitation induced about a 50% increase (Fig. 8i,j). This is not suprising because, unlike the TNF-α treatment, only a small fraction of cells around the periphery of the crater is impacted by mechanical trauma.

**Figure 9:**
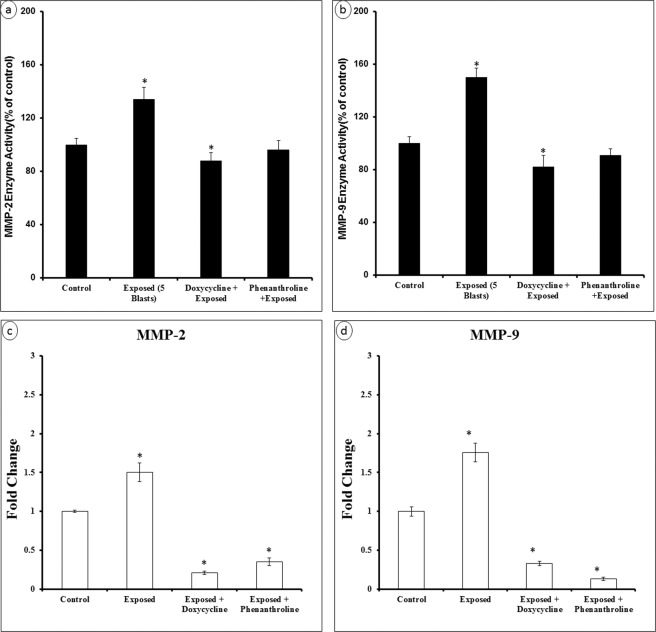
MMP-2 and 9 enzyme activity and gene expression in response to microcavitation. (**a**,**b**) Cells exposed to microcavitation demonstrated significantly increases of the MMP-2 and 9 enzymatic activity when compared to the control. This increased activity was inhibited by pre-treating the cells with either doxycycline or phenanthroline (a general tissue inhibitor of metalloproteinase). (**c**,**d**) Doxycycline or phenanthroline downregulated the MMP-2 and -9 gene expressions. Data represent mean ± SD of 3 independent experiments. *p < 0.05 compared to the control (no treatment).

**Figure 10:**
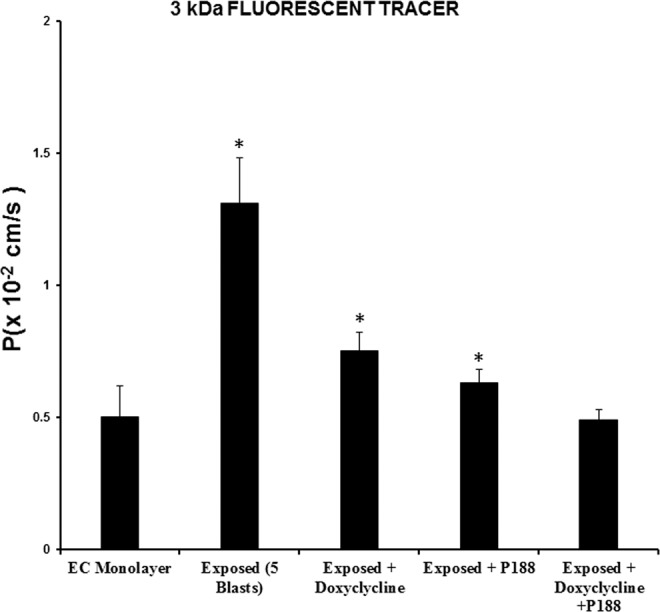
P188 and MMP blocker attenuate the permeability. Permeability was significantly increased in cells exposed to microcavitation when compared to the control (i.e., EC monolayer). The increased permeability is inhibited by P188 or doxycycline. The combination of P188 and doxycycline was shown to further restore the permeability to that observed in control cells and was indistinguishable (p > 0.05). Data represent mean ± SD of 6 independent experiments (n = 6). *p < 0.05 compared to the control (no treatment).

To examine further how P188 might inhibit MMP-2 & 9, we measured the MMP-2 & 9 activities and gene expressions after treating the cells with doxycycline or phenanthroline. Briefly, primary brain endothelial cells were grown under different conditions- control (no treatment), exposed to microcavitation, treated with doxycycline (20 mM) or with phenanthroline (0.5 mM; membrane permeable, general metalloprotease inhibitor) and then exposed to microcavitation. Endothelial cells exposed to microcavitation significantly increased the MMP-2 & 9 enzymatic activity when compared to control. The increased activity was negated with doxycycline pretreatment (Fig. 9a,b). To further validate the results, the gene expressions were also quantified to demonstrate the decreased MMP activities were consistent with down-regulation of the two genes (Fig. 9c,d). Because phenanthroline is a general tissue inhibitor of metalloproteinases (TIMPs), the inhibitory effect on the MMP-2 & 9 activities is less pronounced than doxycycline, as expected. Nonetheless, treatment of mechanically injured cells with a non-specific general metalloproteinase inhibitor was determined capable of decreasing the enzymatic activities of MMP-2 & 9. Primary brain endothelial cell monolayers exposed to microcavitation displayed a significant increase in the permeability of tracer molecules (see Fig. 4). The role of MMPs in increasing the permeability was therefore probed and determined. Following a microcavitation event, doxycycline was shown to reduce the permeability (Fig. 10). However, it was not as efficient as treating the cells with P188. Interestingly however, the combination of doxycycline and P188 together was observed to essentially restore the permeability to the control level, suggesting that P188 could inhibit intracellular signaling pathways other than those affected by doxycycline, or P188 and doxycycline together negate the disruption of tight junctions.

**Figure 11:**
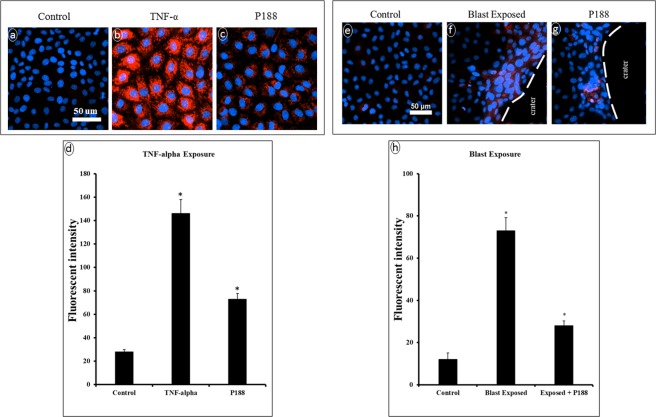
Determination of superoxide levels. (**a**,**d**) In control cells, no fluorescently detectable signals of superoxide (MitoSoz, red) were recorded. **(b)** Inflammatory brain endothelial cells show high levels of superoxide in response to TNF-α. **(c)** P188 treatment noticeably diminished the superoxide level. (**e**) Microcavitation increased the superoxide level especially in the cells located around the periphery of the crater (denoted by dotted line). (**g**) The superoxide levels in the endothelial cells around the periphery of the crater are noticeably diminished in response to the P188 treatment (0.5 mM for 6 hr post-treatment). Quantitative analysis of superoxide levels in response to TNF-α (**d**) or to microcavitation (**h**). Data represent mean ± SD of 3 independent experiments (n = 3). *p < 0.05 compared to the control (no TNF-α activation nor microcavitation).

Finally, evidence can be found in literature that the NF-kB signaling pathway is highly sensitive to oxidative stress and can promote the MMP expression and activity. Since we have demonstrated the microcavitation-induced upregulation of the superoxide level in multiple cell types including astrocytes and muscle cells, we measured the superoxide levels in brain endothelial cells (Fig. 11). In response to TNF-α, a significant increase in the superoxide level was observed, and P188 was able to reduce such an increase (Fig. 11a through 11d). Similarly, microcavitation also up-regulated the superoxide level. As expected, the increases were most noticeable in the cells around the periphery of a crater (e.g., area of detached cells). P188 was also observed to cause a substantial reduction in the superoxide level (Fig. 11e through 11h). P188 down-regulated ROS in response to either a chemical or mechanical trauma,

## Discussion

Traumatic brain injury can be induced by blunt force to the head, chemical factors and also shockwaves from explosive blasts. Through multiple papers recently published^21,22^, we demonstrated the capability to generate shockwaves using a customized two-electrode system. The formation of micron-size bubbles and the subsequent collapse of the microbubbles were described in detail. We coined the term microcavitation to describe the process and was able to quantitatively study the interaction between brain endothelial cells and the collapse of highly pressurized microbubbles. The collapse of microbubbles (~55 kPa; ~10 s interaction time) was observed to detach cells from the substrate and created a region that is devoid of cells^35^. The area of cell detachment has been referred to as a crater. Examples of the crater have been shown in Figs. 3 through 5. Because TBI typically leads to activation of inflammatory responses and release of free radicals which, in turn, could cause necrosis and eventual apoptosis^21^, we used tissue engineering approaches to develop a model to examine the altered biotransport properties of the blood-brain endothelium and identified key molecules that contributed to dysfunctional endothelium.

There are several models of blood-brain barrier disruption but very few, if any, focuses on the mechanism(s) responsible for blood-brain endothelium disruption. Our model allows us to measure the permeability that might have been modulated due to microcavitation. Using two different molecular weight Dextran, the permeability coefficients under various experimental conditions were measured (see Fig. 4). We recognized that, since microcavitation created a region of detached cells, diffusion of Dextran molecules should be primarily through the crater. It is difficult then to delineate the measured permeability coefficients and determine the contribution to the increased permeability that is associated with disorganized tight junctions. This challenge was resolved by a unique engineering design in which a substrate was physically blocked, except the molecules were allowed to diffuse through the crater. The measured permeability coefficients were then adjusted for the unhindered diffusion through the crater. Such an adjustment reduced the permeability of the smaller 3 kDa Dextran molecules by almost ~3-fold (Fig. 4). However, the adjusted permeability coefficient was nonetheless significantly greater than the permeability through unexposed blood-brain endothelium. When the same experiments were carried out using 10 kDa Dextran, the adjusted permeability coefficient did not differ from the control value. One plausible interpretation would be that even though we observed tight junction disruption, larger molecules are still excluded from diffusing through the disorganized tight junctions. In contrast, diffusion of the smaller molecules (e.g., <3 kDa) appears to be enhanced through tight junctions. Since toxins in the blood are typically small molecules^6,36^, they can more easily cross the brain endothelium through the disrupted tight junctions^6,37^. Eventually, it might lead to thickening of the capillary basement membrane, degeneration of small cerebral arteries, and defective regulation of cerebral blood flow.

Traumatic brain injury causes activation of inflammatory responses and release of free radicals, which leads to necrosis and eventual apoptosis^21^. In response to either chemical (TNF- α) or mechanical (microcavitation) trauma, the MMP expressions are significantly up-regulated. Moreover, the tight junction formation and function appear to inversely correlate with the MMP expressions. Interestingly, our results indicate treating the disrupted endothelial cells for 3 hours with P188 promoted tight junction recovery by down-regulating MMP-2 & 9 and up-regulating ZO-1. A few research groups have also shown that P188 can be internalized and affect cellular activities such as minimizing the inflammatory factors, excess activation of cytokines as well as the release of free radicals^38–40^. Results from our study are consistent and support the therapeutic effects of P188. For example, the tight junction protein (ZO-1) in BECs exposed to microcavitation was suppressed, while a treatment of the injured cells with P188 showed upregulation of the tight junction protein (Fig. 6). The beneficial effects of P188 are reproducible and statistically significant. The mechanism(s) that mediate such effects still remain to be fully elucidated, however. A few studies suggest that P188 might inhibit the NF-kB signaling pathway^41^. If validated, it would provide a clearer understanding of the interaction between P188 and NF-kB that is a known transcription factor for MMP expressions^33,42,43^. In an animal model study, Wang *et al*.^39^ demonstrated that P188 exerts neuroprotective effects by preventing MMP-mediated tight junction degradation. In a stroke animal model study, P188 has again been shown to provide protection against cerebral ischemia through inhibition of MMP-9^44,45^.

**Figure 12:**
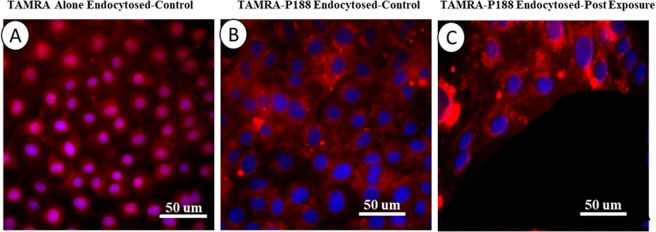
Internalization of P188. **(A)** Image of internalized TAMRA fluorophores (red) alone in control cells. Nuclei counterstained with DAPI (blue). The fluorophores diffused into the cytoplasm and nuclei. **(B)** Immunofluorescent images showing endocytosis of conjugated TAMRA-P188 complexes in control cells. Diffusion of the complex into the nuclei was not evident. **(C)** Immunofluorescent images showing endocytosis of conjugated TAMRA-P188 complexes in cells exposed to microcavitation. It is evident that the complexes diffused into the nuclei in some cells (e.g., purple color), suggesting potential reorganization of the nuclear envelope caused by microcavitation.

It still remains to be fully elucidated how P188 interferes with downregulation of MMPs. In addition to the plausible coupling mechanism to the NF-kB signaling through down-regulation of ROS, we speculate there might be an alternative. As a first step toward a better elucidation, we successfully conjugated P188 with a fluorescent dye (TAMRA) and incubated BECs with the conjugated P188. A shown in Fig. 12, the TAMRA dye alone was able to diffuse through the cell membrane and, in some cells, it appears to have penetrated into the nucleus (Fig. 12A). When the conjugated TAMRA-P188 complex was introduced to BECs, P188 was shown to be internalized in control cells but excluded from the nucleus (Fig. 12B). In response to microcavitation, the cells on the periphery of the crater show an increased fluorescence intensity. It may be likely due to an influx of the complexes through the compromised cell membrane. Further, we did observe in some cells that the P188-TAMRA complexes penetrated inside the nuclei of the injured cells, suggesting compromised nuclear transport function. Although more systematic studies are indeed required to reveal the molecular interactions that involve P188 in the injured cells, it appears plausible that P188 can be coupled to the intracellular signaling pathways as well as in yet to be defined nuclear interactions.

Maintenance of the BECs relies on the interaction of endothelial cells and basement membrane. Together, they play an essential role in the biotransport of molecules from the blood to the brain and vice versa. TBI is typically accompanied by an increased permeability of the BBB. Studies have shown that an increased level of MMP-2 & -9 plays a key role in the BBB destruction by induction of inflammatory cascade reaction^46^. The activity of the two proteolytic enzymes is associated with the BBB permeability^47^ and facilitates degradation of tight junction proteins. Conversely, inhibition of MMP-2 & -9 promotes recovery of the BBB^48^. Therefore, targeting MMP-2 & -9 may be one of the strategies for the treatment of TBI. Our results show that the excessive expression of MMP-2 & -9 can be reversed by P188 treatment in response to an inflammatory (Fig. 7) or mechanical trauma (Fig. 8). As a confirmatory study, doxycycline was applied to diminish the enzymatic activates of MMP-2 & 9. Microcavitation-induced upregulation of both the MMP activities and gene expressions were shown to be mitigated by treating the cells with doxycycline (Fig. 9). We further explored the effect of doxycycline to examine the permeability through tight junctions (Fig. 10). As expected, doxycycline not only inhibited the upregulation of MMPs but also significantly restored the permeability. It should be mentioned that when the combination of doxycycline and P188 was applied together, the increased permeability of the brain endothelium that was attributed to microcavitation was restored to the control value. These *in vitro* data provide convincing evidence that P188 along with an adjuvant therapeutic compound could offer a viable treatment for TBI.

## Conclusion

**Figure 13:**
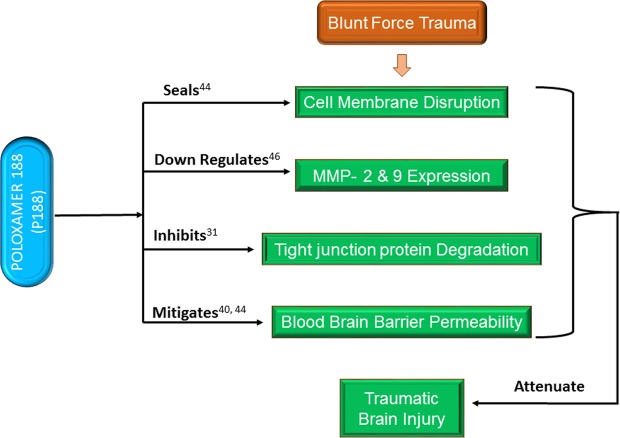
Schematics showing the potential mechanisms underlying the protective effects of P188. Blunt force trauma increases ROS. The oxidative stress-sensitive signaling pathways (e.g., NF-kB) can up-regulate MMP- 2 & 9 in the brain endothelium which, in turn, degrades the tight junction structure and integrity. The brain endothelium transport properties are modulated and lead to an increased BBB permeability. The BBB protection by P188 against traumatic brain injury is proposed to be mediated by resealing the compromised cell membrane, decreasing the ROS levels, down-regulating MMPs, which inhibits the degradation of tight junction, and thereby restores the biotransport properties of the brain endothelium. Collectively, P188 attenuates the extent of injuries caused by blunt force brain traumas.

In summary, we established and characterized an *in vitro* brain endothelium model using a biopolymeric membrane. Using the model, changes in the biotransport properties were experimentally quantified in response to a mechanical trauma such as microcavitation, and the potential restorative effects of P188 were established. We determined the role of P188 for repairing the injured endothelium in response to an inflammatory or mechanical trauma. Although the specific molecular mechanism(s) mediating the therapeutic effects of P188 are under investigation, such effects are likely found at the level of the cell membrane, cytoplasm, between cells, and even in the nucleus. Based on our collective findings from the study, we postulate and propose a working model that P188 delivers therapeutic effects at multiple levels (Fig. 13). P188 reseals the disrupted cell membrane, downregulates MMPs through suppressing ROS, and subsequently restores the structural and functional integrity of tight junctions. This leads to at least partial restoration of the biotransport properties of the brain endothelium. These multi-level beneficial effects of P188 are expected to attenuate the extent of traumatic brain injury. Finally, the model we developed and used in this study is quite simple, easy to engineer, convenient, and reproducible to provide a platform for studies of molecular mechanisms and rapid screening for potential therapeutic compounds.

## Materials and Methods

### *In vitro* brain endothelium model

Mouse brain endothelial cells were cultured on a synthetic polyester terephthalate (PETE) membrane (Sterlitech, Kent, WA) that contains 1 μm diameter pores at the density of 2 × 10^7^ pores /cm^2^. This well-defined membrane allowed us to establish a monolayer of endothelial cells and determine the endothelium permeability. Balb/c mouse primary brain microvascular endothelial cells (MPBMECs; Cell Biologics Inc., Chicago, IL) were grown in endothelial basal medium-2 (EBM-2) with EGM-2 kit (Lonza, Walkersville, MD) in a flask coated with a gelatin-based coating solution. Cells were seeded at a density of 6.60 × 10^4^ cells/cm^2^ on the PETE membrane coated with fibronectin (l μg/ml). To confirm the viability of MPBMECs, an *in vitro* angiogenesis was performed. Briefly, reduced growth factor Matrigel (200 uL; Corning, Corning, NY) was pipetted onto a 22 × 22 mm coverslip in a petri dish and incubated at 37 °C and 5% CO_2_ for 30 min to solidify Matrigel. Once Matrigel was set, approximately 5 × 10^4^ cells MPBMECs were seeded on the gel and tube formation was observed after 6 hrs. To accurately visualize the tube formation, FITC green cell tracker was added to the cells before seeding.

### Microbubble exposure chamber

A detailed description of the simulated blast and microbubble chamber used in this experiment has been provided elsewhere^21,22^. This chamber is designed to fit the microscope stage for real-time imaging. Briefly, two platinum electrodes were symmetrically embedded in the middle of the chamber (~10 mm high; Fig. 1a). Shockwaves were generated by applying an electrical pulse across the electrodes that were separated by 700 um. Temporary electrical breakdown of water creates shock waves, followed by the generation of microbubbles. Potential artifacts such as temperature rise, excessive-high electric field, and the impact of shock waves were all carefully assessed, measured, and addressed previously. For example, a temperature rise of <0.5 °C was measured^21^, and the peak shock wave pressure was recorded 10 MPa^28^. The pressure of collapsing microbubbles was also measured to be 55 kPa^28^, which is at least two orders of magnitude smaller than the peak pressure but certainly large enough to adversely impact the cells at or near the site of microcavitation. The principle of symmetry was applied to study the effect of shock waves by plating cells at the bottom of the chamber (Fig. 1a). Should the shock waves (0.3 us travel time across the depth of the chamber) cause any measurable impact, these effects should be observed both at the top and bottom of the chamber. The cells plated at the bottom of the chamber were observed viable and functional and expressed, for example, the baseline fluorescent markers of apoptosis^21^. In contrast, the microbubbles rose only to the top of the chamber and collapsed onto the plated cells. The density of microbubbles, their speed, and size distribution following propagation of shock waves were recorded, analyzed, and reported in our previous study^21^. The cellular and molecular signatures that are associated with endothelial cell damage are yet to be determined.

### Immunostaining for ZO-1, Actin, and MMP-2 & 9

Tight junctions in the brain endothelial cells were visualized using zonula occludens 1 (ZO-1) monoclonal antibody conjugated with Alexa Fluor 488 (Thermofisher Scientific, Waltham, MA). The BECs were fixed with 4% paraformaldehyde in PBS for 10 minutes at room temperature and blocked with 3% of BSA for 1 hour. Cells were incubated with fluorescently conjugated ZO-1 monoclonal antibody for 1 hour and kept in the dark. Nuclei were stained with DAPI at a dilution of (1:1000). For MMP-2 & 9 analyses, cells were fixed with 4% paraformaldehyde in PBS, permeabilized with 0.1% Triton X-100 for 3 minutes and blocked with 3% of BSA for 1 hour. Cells were incubated with the MMP-2 & 9 primary antibodies (1:500, 4.3 μg/ml, Santa Cruz Biotechnology, Santa Cruz, CA) and subsequently incubated with Alexa Fluor 488-conjugated goat anti-rabbit IgG (1:1000, 2 μg/ml, Santa Cruz Biotechnology).

### RNA isolation and gene expression measurements

Total RNA was isolated according to protocols supplied by the manufacturer (Quick-RNA Microprep Kit, Zymogen Research Corp, Irvine, CA). Briefly, cells were lysed in an RNA lysis buffer washed with 95% ethanol and RNA prep buffer, and then sequentially washed with RNA wash buffer, and RNA was eluted with 15 ul of DNase/RNase-Free water. The sample was treated with RQ1 RNase-Free DNase from (Promega Life Sciences, Madison, WI) to remove any remaining DNA. NanoDrop Spectrophotometer was used to measure the 260/280 ratio as well as RNA yield (quantity, ng/ul). RT-PCR was carried out using the manufacturer’s protocols. cDNA was synthesized using AzuraQuant cDNA Synthesis kit (Azura Genomics, Raynham, MA). The mastermix was prepared using the Green Fast qPCR Mix Hirox Kit (Azura Genomics). Two microliters of cDNA were used in each subsequent PCR reaction. Specific primers for BBB tight junction protein; zonula occludens 1 (ZO-1), and a housekeeping gene, GAPDH (RealTimPprimers.com), were designed and synthesized according to published sequences (RealTimePrimers.com). PCR amplifications were performed in a final volume of 20 ml of the mastermix using AzuraQuant Green Fast qPCR Mix Hirox as instructed by the manufacturer. Using applied biosystems 7300 Fast Real-Time PCR system (Thermo Fisher Scientific), amplifications were done for 45 cycles using a denaturation step at 95 °C for 5 s, an annealing step at 60 °C for 25 s, and polymerization step at 72 °C for 45 s.

### Western blots

Endothelial cells were washed twice with PBS and lysed with RIPA buffer (Sigma) plus protease inhibitor cocktail (Sigma). Protein concentration was quantified via Coomassie Protein Assay Reagent (Thermos Scientific), and proteins were then resolved by Mini-PROTEAN TGX Stain-Free Gel (BIO-RAD) before transferring to 0.2 um PVDF membrane in Trans-BLOT Turbo Transfer Pack (BIO-RAD) using the Trans-Blot Turbo Transfer Stater System Mini. Blocking was conducted for 1: 30 h in Tris-buffered saline (10 mM Tris-HCl, 100 mM NaCl, pH7.5) containing 0.1% Tween-20 (TBST) and 5% BSA. Samples were probed overnight at 4 °C with rabbit polyclonal ZO-1 tight junction protein antibody (1:2000, Abcam) and GAPDHS rabbit polyclonal antibody (1:1000, Proteintech) diluted in TBST with 5% BSA. After being washed three times for five minutes with TBST, the sample was incubated with goat anti-rabbit IgG H&L (HRP) (Abcam) for 1 h at room temperature. The membrane was washed three times for five minutes with TBST before developing/enhancing the signal using Clarity Max Western ECL Substrate. The image was acquired using ChemiDoc Touch MP Imaging System and analyzed with Image Lab Software.

### MMPs activities assay, fold change, and permeability measurement

EnzChek MMP-2 & 9 Assay Kit were used for MMP-2 & 9 activity assays. Cells were cultured in endothelial cell complete media and divided into the following groups: control (no treatment), exposed (5 blasts), doxycycline (20 mM) + exposed (5 blasts), doxycycline (20 mM), and phenanthroline (0.5 mM). Before initiating experiments, cells were washed with PBS, and no serum media was applied. Dishes in the treatment groups were pretreated with doxycycline or phenanthroline for 2 h at 37 °C. Cells were exposed to microcavitation and incubated at 37 °C for 1 h. All groups were homogenized with lysis buffer containing 0.1% Triton-X 100, and protein was estimated with Pierce BCA Protein Assay Kit. Equal amounts of protein from each sample were aliquoted in lysis buffer. Samples were then analyzed using a plate reader at excitation 495 nm and 515 nm to quantitate MMP-2 & 9 activities. Primary brain endothelial cells were grown under the conditions described above. At the end of all experimental conditions, RNA from the cells was extracted and used for PCR analysis of the MMP-2 & 9 gene expressions. Finally, the permeability of the brain endothelium model exposed to doxycycline, P188, or in combination of them both was measured.

### *In vitro* diffusion model and permeability experiment

The permeability experiments were carried in a 2-step process (Fig. 1b–d). After seeding and culturing cells on a portable insert, it was transferred to the microbubble exposure chamber (see above). Following a microcavitation event, the insert was then placed in a custom-designed diffusion chamber. The insert that contains the endothelial cells was mounted into the chamber, and its edge was sealed to prevent leakage. With the insert properly secured in the upper chamber, it was then filled with PBS containing FITC Dextran molecules. The lower chamber was filled with 5 ml of PBS without phenol red. The permeability experiment was conducted in the dark and at room temperature. FITC Dextran of molecular weight 3 or 10 kDa were used for the experiment. A small volume of 25 µl was collected from the lower chamber at one-hour intervals for 6 hours and stored at −80 °C. Fluorescence intensities were measured using a fluorescence plate reader (Synergy HT). Fluorescent intensity calibration standard was obtained by serial dilution of fluorescent Dextran. Concentrations in the collected samples were determined from the calibration curve. The Permeability coefficient was then calculated by modifying the equation from Li *et al*.,^49^; $P=\frac{[({\bf{C}}2{\bf{a}}\,-\,{\bf{C}}1{\bf{a}})\,\ast {\bf{V}}{\bf{a}})]\,}{[\,{\bf{A}}\ast \varDelta {\bf{t}}\ast \,{\bf{C}}{\bf{o}}]}$, where P is the permeability coefficient, C_2a_ & C_1a_ are the concentration in the abluminal chamber at different time intervals, V_a_ is volume of the abluminal chamber, A is the surface area of the membrane, ∆t is the duration of steady-state flux, C_o_ is the concentration in the luminal chamber. The expression (C_2a_ - C_1a_)/∆t is considered as the slope of the diffusion curve over time.

### Dose-dependent study of P188

Dose-dependent study on the proliferation of primary brain endothelial cells was measured using CellTiter 96® AQueous One Solution Cell Proliferation Assay for 24 and 48 hours. It is a colorimetric method for determining the number of viable cells in proliferation or cytotoxicity assays. It is non-toxic, which allows an extended period of incubation. Briefly, cells were incubated with CellTiter 96® AQueous One Solution Cell Proliferation Assay for an hour. Absorbance was recorded at 490 nm using a plate reader, and the plate returned to an incubator until 48-hour time point. Untreated cells were used as control. The viability of cells following a 24-hour exposure to P188 was assessed with the MTT assay. Briefly, after exposure of BECs to P188, cells were treated with MTT (5 mg/ml) for 1 hour at 37 °C. The formazan product generated was solubilized by addition of 20% sodium dodecyl sulfate and 50% N, N-dimethylformamide, and quantified by measuring its absorbance at 490 nm. Resulting sample absorbance was used to calculate cell viability.

### Statistical analysis

All experiments were replicated at least three times. Data are presented as mean ± standard deviation (SD) and compared using a one-way ANOVA followed by a Tukey’s test for post hoc analysis. p < 0.05 was indicated with an asterisk (*).

## Supplementary information

Supplementary information.
