# Author Correction: Modulation of *in vitro* Brain Endothelium by Mechanical Trauma: Structural and Functional Restoration by Poloxamer 188

**DOI:** 10.1038/s41598-020-62005-y

**Published:** 2020-03-18

**Authors:** Edidiong Inyang, Vinay Abhyankar, Bo Chen, Michael Cho

**Affiliations:** 1 Department of Bioengineering, University of Texas at Arlington, Arlington, TX United States; 2 Department of Biomedical Engineering, Rochester Institute of Technology, Rochester, NY United States

Correction to: *Scientific Reports* 10.1038/s41598-020-59888-2, published online 20 February 2020

The Acknowledgements section in this Article was omitted. The Acknowledgements section should read:

“This work was supported by a grant (N00014-16-1-2140) from the Office of Naval Research. Edidiong Inyang was a Louis Stokes Alliance for Minority Participation (LSAMP) Fellow”.

