# Supplementary material for: Modulation of in vitro Brain Endothelium by Mechanical Trauma: Structural and Functional Restoration by Poloxamer 188
Source: Sci Rep. 2020 Feb 20;10:3054. doi: 10.1038/s41598-020-59888-2 (PMC7033190; doi:10.1038/s41598-020-59888-2)
Supplement: Supplementary file 1 — Supplementary information. [file 41598_2020_59888_MOESM1_ESM.docx]

**Modulation of *in vitro* Brain Endothelium by Mechanical Trauma:**

**Structural and Functional Restoration by Poloxamer 188**

Edidiong Inyang^1^, Vinay Abhyankar^2^, Bo Chen^1^, and Michael Cho^1^

^1^Department of Bioengineering, University of Texas at Arlington, Arlington, TX.

 ​

^2^Department of Biomedical Engineering, Rochester Institute of Technology, Rochester, NY

**Supplementary Figure**

**
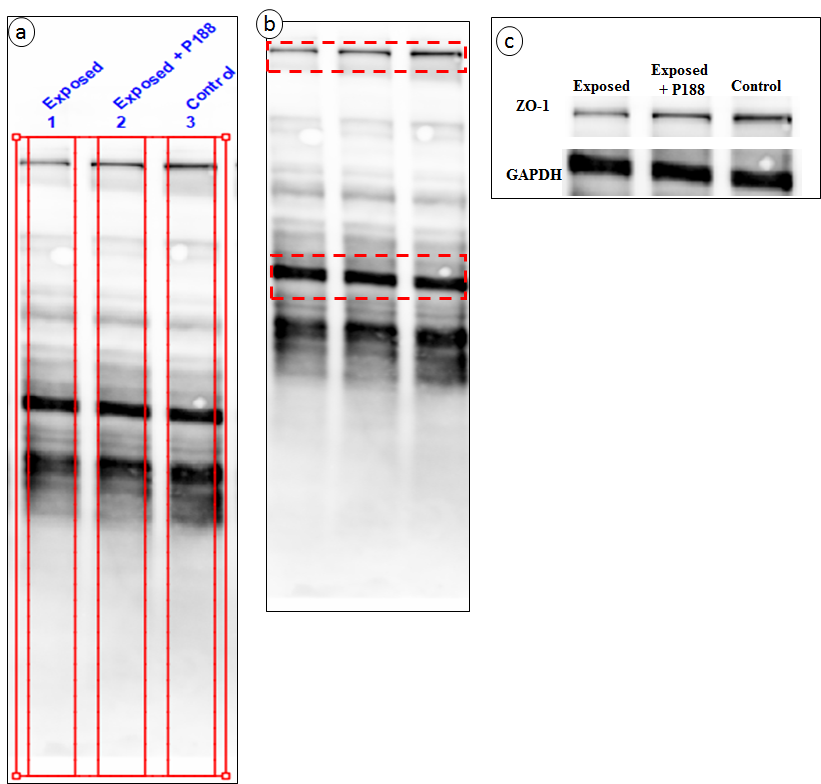
**

**S1**

**Supplementary Figure Legend**

**S1:** Western blot analysis after treatment with P188. (**A**) Full-length Western blot analysis using Image Lab 6.0.1. (**B**) Full-length Western blot analysis from (**A**) highlighting the area of interest in red dotted box. (**C**) Area of interest cropped from full-length Western blot analysis.
